# Machine learning in knee injury sequelae detection: Unravelling the role of psychological factors and preventing long‐term sequelae

**DOI:** 10.1002/jeo2.70081

**Published:** 2024-11-22

**Authors:** Clément LIPPS LENE, Julien Frere, Thierry Weissland

**Affiliations:** ^1^ Université de Bordeaux, Laboratoire IMS, UMR 5218, PMH_DySCo Pessac France; ^2^ Univ. Grenoble Alpes, CNRS, Grenoble INP, GIPSA‐Lab Grenoble France

**Keywords:** force‐velocity, knee injury, machine learning, rehabilitation, sequelae detection

## Abstract

**Purpose:**

This study evaluated the performance of three machine learning (ML) algorithms—decision tree (DT), multilayer perceptron (MLP) and extreme gradient boosting (XGB)—in identifying regular athletes who suffered a knee injury several months to years prior. In addition, the contribution of psychological variables in addition to biomechanical ones in the classification performance of the ML algorithms was assessed, to better identify factors to get back to competitive sport with the lowest possible risk of new knee injury.

**Methods:**

A cohort of 96 athletes, 36 with prior knee injuries, practicing an average of 5.7 ± 2.4 h per week, participated in a horizontal force‐velocity test on a ballistic ergometer providing data of force, velocity and power from each lower limb. They also completed a psychological questionnaire, which included components from the Knee Injury and Osteoarthritis Outcome Score (KOOS) and the Sport Anxiety Scale (SAS). The three ML algorithms were trained on a thousand different train‐test sets. Also, Shapley values were calculated for each input variable of a data set to highlight its contribution to the prediction from an ML model.

**Results:**

Over a thousand cross‐validations, higher area under the curve (AUC) values were obtained when accounted for the psychological attributes (*p* < 0.001). Also, higher AUC values were obtained from MLP compared to XGB or DT (*p* < 0.001). XGB exhibited higher AUC values than DT (*p* < 0.001).

**Conclusions:**

Our results suggested that psychological factors play a more important role in recognition than biomechanical factors, with KOOS and SAS scores ranking high in the list of influential factors. Additionally, the computing stability of MLP could be recommended for classification tasks in the context of knee injuries.

**Level of Evidence:**

Level III.

**Abbreviations**
ACL: anterior cruciate ligament
AUC: area under the curve
BMI: body mass index
DT: decision tree
KOOS: Knee Injury and Osteoarthritis Outcome Score
ML: machine learning
MLP: multilayer perceptron
QOL: quality of life
RTP: return to play
SAS: Sport Anxiety Scale
SHAP: shapley value method
XGB: extreme gradient boosting

## BACKGROUND

Knee injuries, particularly anterior cruciate ligament (ACL) injuries, are among the most common in sports [10, 32] and the rate at which they occur has considerably increased [15, 20]. The return to play (RTP) after an ACL reconstruction is complex with only 44%–65% of athletes returning to their previous level of play [6, 35]. Despite the complexity of these injuries, most studies have focused on a limited set of risk factors and often overlook the significant role of psychological factors [9]. For example, 66% of athletes did not return to competitive sports after rehabilitation due to fear of reinjury [3]. Thus, to better understand ACL injury risks, it is crucial to consider psychological factors alongside biomechanical ones. Recent studies also highlighted the force–velocity relationship as a tool for both injury prevention and optimizing ACL rehabilitation, especially by addressing strength asymmetries at different velocities [8, 31].

Machine learning (ML) algorithms are effective at analyzing large volumes of data helping to identify patterns and predict knee injury risk by considering simultaneously various physical, biomechanical and psychological factors [10, 18, 33]. Decision trees (DT) are nonparametric supervised learning algorithms that adopt a tree structure comprising nodes (attributes of a database) and branches (decision based on attributes). Such tree‐based ML algorithms are frequently used for injury prediction due to their interpretability. As usually done in ML, a Receiver Operating Characteristic (ROC) curve is used to graphically represent the performance of a binary classification model, and the area under the curve (AUC) quantifies the ‘quality’ of classifier predictions, which serves as a key performance measure [7]. However, DTs often overfit the training data, leading to poor generalization [16]. More advanced methods like extreme gradient boosting (XGB), which builds strong predictive models through a series of DTs, and multilayer perceptron (MLP), a type of neural network that processes data through multiple layers, offer improved performance and stability [16]. As recommended by Tjønndal et al. [33] and similarly to DT for which decision rules are displayed on the tree structure, XGB and MLP predictions could be explained with the use of the Shapley values (SHAP) [30]. The SHAP method further enhances model interpretability by explaining the contribution of each input to each model prediction, enabling personalized identification of knee injury risk factors in addition to general population risk factors, as highly recommended by Van Eetvelde et al. [34]. The purpose of this study was to investigate and compare the predictive performance of DT, XGB and MLP methods in identifying knee injury sequelae. We hypothesized that (i) XGB and MLP would outperform DT in recognizing knee injury sequelae and provide more accurate identification of injury risk factors on both the global and individual levels using the SHAP method and (ii) the inclusion of both biomechanical and psychological variables in the models would enhance performance compared to models relying solely on biomechanical factors. Our objective was for the ML models to accurately identify individuals with a history of knee injury, rather than predicting injury occurrence, as previously done [18]. This approach would help prevent the recurrence of knee injuries and improve RTP by detecting athletes who still have injury sequelae, enabling the creation of tailored rehabilitation sessions to optimize their return to competitive sports.

## METHODS

### Participants

**Figure 1:**
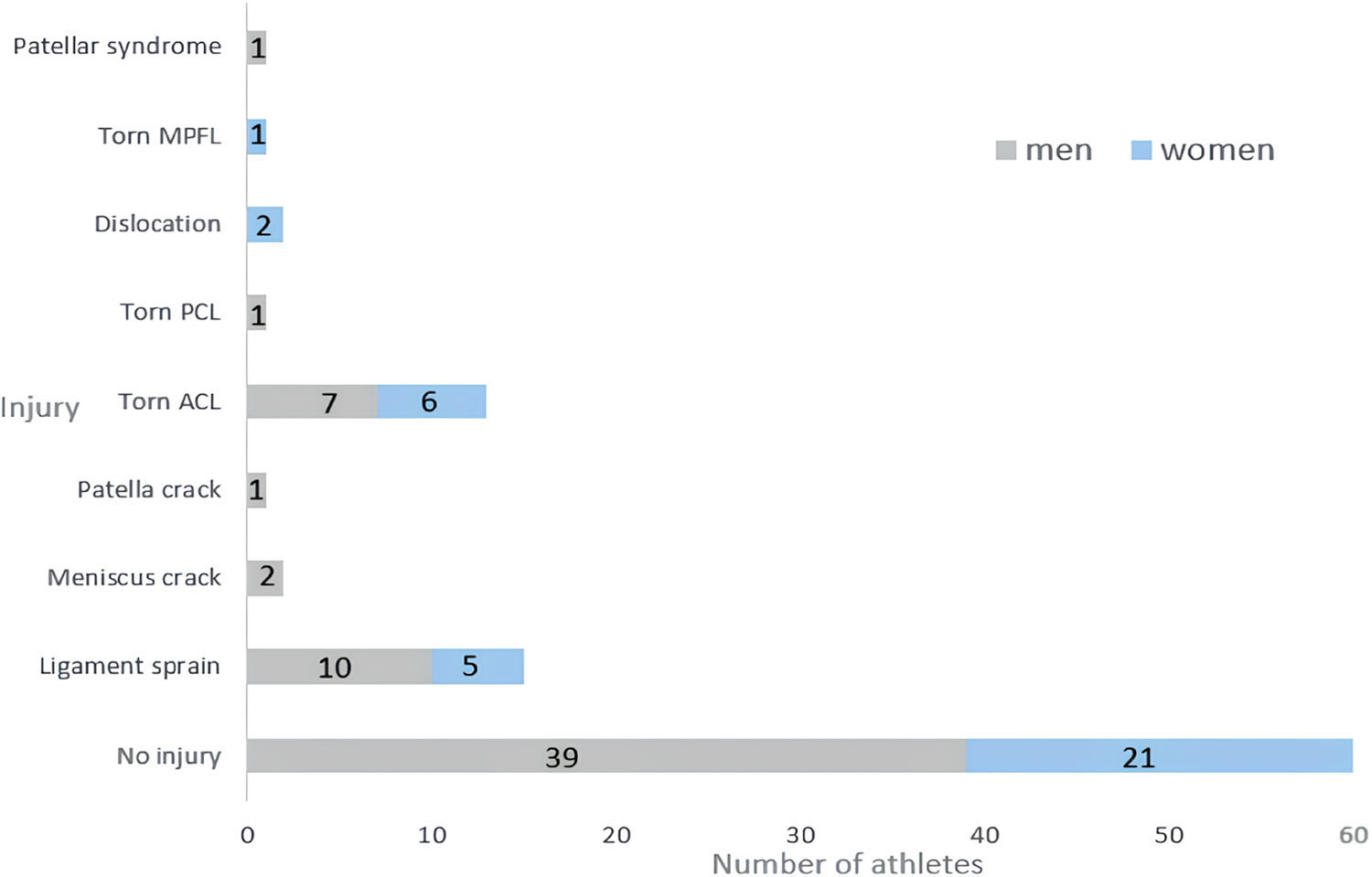
Injury distribution in the cohort of 96 athletes that participated in the study. ACL, anterior cruciate ligament; MPFL, medial patellofemoral ligament; PCL, posterior cruciate ligament.

To address these hypotheses, a database consisting of 96 athletes has been constructed. The data set consisted of 35 women with an average age of 21.1 ± 1.4 and 61 men with an average age of 21.9 ± 2.8. To conduct the participant selection, the following inclusion criteria were used: practicing sport at least once a week and in case of a previous knee injury, being in the last stages of rehabilitation. Indeed, every participant was at least a recreational athlete and the average time of practice by week was 5.7 ± 2.4 h. Noninclusion criteria were as follows: ongoing knee pain, ongoing muscle injury or being within the first 6 months of rehabilitation after an ACL tear. As a consequence, the sample included athletes in good physical condition without a history of knee injury (*n* = 60, 39 men and 21 women) and athletes in good physical condition but with a history of knee injury or in the final stages of the process of RTP after a knee injury (*n* = 36, 22 men and 14 women). Finally, whatever their condition, no individuals expressed having felt pain during the experiment. In the case of multiple knee injuries, only the most serious one was reported (Figure 1). The most represented sports in the cohort were rugby (21 men, three women), football (13 men, four women) and judo (six men, six women). All participants signed written consent, and the study was approved by an ethics committee (CERSTAPS: IRB00012476‐2022‐21‐01‐149).

### Procedure

For each participant, the experimental session began with the completion of an informational questionnaire and an 8‐min warm‐up, which included cycling, squats and squat jumps. This was followed by a minimum of 10 unloaded pushes on the ballistic ergometer to familiarize participants with the supine position required for the test. During both the warm‐up and recorded session, participants were instructed to apply their force as quickly as possible on the force plates, with one force plate positioned under each foot [22], while verbal encouragements were provided. The horizontal force–velocity test started with ballistic push‐offs performed in a supine position on a frictionless cart. The test included five load conditions: 0%, 30%, 60%, 90% and 110% of the participants' body weight, performed in a random order [21]. For each load level, two horizontal push‐offs were performed with a 30‐s interval. A 2‐min rest period was provided between each load change to prevent fatigue. The ergometer was the same as the one presented by Macchi et al. [22]. It minimized the risk of injury as it was a guided movement and prevented loaded landings.

### Data collection

The informational questionnaire consisted of questions providing basic information on the athlete and their sports practice, psychological questions from the Sport Anxiety Scale (SAS) and the Knee Injury and Osteoarthritis Outcome Score (KOOS). Both SAS and KOOS provided an overview of athletes' anxiety levels prior to competition and of their perception of their knees, respectively. Higher values indicated lower anxiety and better knee perception [4, 23, 24]. Two force plates [Kistler 9260AA3 (0.5 × 0.3 m)] were used to record the components of the ground reaction forces produced under each foot with a sampling frequency set at 2 kHz. Additionally, the displacement of the cart caused by the push‐off movement performed by the athlete was recorded with a linear encoder of 0.1 cm accuracy (Micro‐Epsilon WDS‐3000‐P115‐SR‐U).

### Data preprocessing

The first preprocessing step involved extracting the biomechanical variables from the sensor measurements with Matlab scripts (MATLAB and Statistics Toolbox Release 2022a, The MathWorks, Inc.). For each push‐off, the force and displacement data were retained during the push‐off phase (i.e., when participants began to apply force on the force plates until take‐off). From these retained raw data, the script computed 20 variables (also called attributes) that depicted the biomechanical signature of the push‐off (Table 1). In addition to these biomechanical variables, nine others came from the informative questionnaire, while five psychological variables were retained (Table 1). Although these biomechanical variables were not specific to the knee, they were similar to other physical tests (e.g., hop test) used to assess the RTP of an injured athlete [26]. Therefore, these selected biomechanical variables provided information on the athletes' capacity to develop high force, power and velocity as required in several sport movements.

**Table 1:** All variables extracted from the questionnaires (informative and psychological) and push‐off biomechanics.

Feature	Unit
History of knee injury	1 if yes, else 0
Height	cm
Body mass	kg
Body mass index	kg.m^−2^
Age	years
Gender	unitless
Sport practiced	unitless
Amount of practice	hours/week
Level of practice	unitless
SAS score	unitless
KOOS symptoms	unitless
KOOS sport and recreation	unitless
KOOS pain	untiless
KOOS quality of life	unitless
Total peak force	N
Left peak force; right peak force	N
Total mean force	N
Left mean force; right mean force	N
Mean velocity	m.s^−1^
Total mean inertial force	N
Left mean inertial force; Right mean inertial force	N
Push‐off time	s
Inertial push‐off time	s
Push‐off distance	m
Total effective force ratio	unitless
Left effective force ratio; Right effective force ratio	unitless
Time to reach total peak force	s
Maximum power	W
Force at maximum power	N
Velocity at maximum power	m.s^‐1^

Abbreviations: KOOS, Knee Injury and Osteoarthritis Outcome Score; SAS, Sport Anxiety Scale.

Two data sets were built from the cohort to predict the presence of knee injury sequelae. The first one aggregated the load level with attributes listed in Table 1 excluding the KOOS and SAS scores. It contained 964 rows (corresponding to each push for each load level and each athlete) and 30 attributes. The second data set was similar to the first one but included the five scores from the KOOS and the SAS. It then consisted of 964 rows and 35 columns.

In this study, the selected ML algorithms were trained from a training data set, and their generalization was tested on a test data set. The train‐test split of the overall data set followed a standard 80%–20% ratio [14]. To cope with the sample size, a cross‐validation procedure was done, with a thousand subject‐based 80%–20% train‐test splits. In other words, athletes in the training set were not included in the test set [29]. In each of the thousand trials, the athletes in the train and test sets were randomly reassigned while maintaining a consistent distribution of the two classes: athletes with a history of knee injury and those without. We chose a thousand iterations of train‐test splits to preserve computing efficiency, although over a billion combinations were possible. Finally, as a standard practice to achieve optimal performance metrics after training, a min–max normalization was applied to each column of the selected train and test data sets [1, 25].

### Algorithm selection

The DT method is a commonly used ML algorithm and served as the initial classifier in the current study. It is a nonparametric supervised learning algorithm that adopts a tree structure comprising nodes (attributes of our database) and branches (decision based on attributes). DT aims to tightly fit the training data and identify relationships that maximize a chosen classification metric within the data set [14]. The second classifier of the study was the XGB. It works by building and training an ensemble of DT and combining sequentially their predictions to make a final one. Thus, it reduces the risk of overfitting the data and can also achieve better performance [5]. The third classifier of the study was a MLP with one hidden layer containing 30 neurons and the ReLU activation function. The MLP aims to approximate a function that maps an input to a category. The network defines a mapping and learns the values of the parameters that result in the best approximation of the function [11]. In addition, MLPs have a larger number of mathematical parameters than DT and XGB. This means that they require more data to perform well but can capture more complex relationships between them.

For each algorithm, the class imbalance problem in the data set was addressed in the same way, as inspired by King and Zeng's study [19]. Class weights have been computed with the following formula for the i‐th class: $\frac{n_{\mathrm{samples}}}{n_{\mathrm{classes}}\;n_{\mathrm{it}h_{\mathrm{classes}}}}$. These weights were applied during model training, ensuring that prediction errors for athletes with a history of knee injury were given equal importance to errors for athletes without knee injury.

### Metrics

The predictive capacity of the different ML models used in the present study was assessed by an ensemble of performance indicators, referred to as metrics, based on the confusion matrix [12]. The confusion matrix is a cross table that records the number of occurrences between two raters, the true classification (rows) and the predicted classification (columns), as shown in Table 2.

**Table 2:** The theoretical confusion matrix.

	History of knee injury	Healthy
History of knee injury	TP	FN
Healthy	FP	TN

*Note*: Columns correspond to the true classification (ground truth) while rows represent the predicted classification made by the model. The objective is to have a diagonal matrix.

Abbreviations: FN, false negatives; FP, false positives; TN, true negatives; TP, true positives.

Herein, true positives represented athletes with a history of knee injury and predicted as such, false positives represented healthy athletes predicted as having knee injury sequelae, true negatives represented healthy athletes predicted as healthy and false negatives represented athletes who had suffered a knee injury but predicted as healthy.

To quantify the proportion of correct predictions, the **accuracy** was computed as follows:

$$\mathrm{accuracy}=\frac{\mathrm{TP}+\mathrm{TN}}{\mathrm{TP}+\mathrm{TN}+\mathrm{FP}+\mathrm{FN}}.$$

In the case of unbalanced classes (the current data sets had two‐thirds of the healthy participants), additional metrics are needed to conclude the efficiency of a model. The proportion of positive predictions that were truly positive, also called **precision**, was calculated as follows:

$$\mathrm{precision}=\frac{\mathrm{TP}}{\mathrm{TP}+\mathrm{FP}}.$$

The **recall**, also named the true positive rate, was calculated as a measure of the model's ability to recognize all positive observations:

$$\mathrm{recall}=\frac{\mathrm{TP}}{\mathrm{TP}+\mathrm{FN}}.$$

Finally, the **F1‐score**, aggregating **precision** and **recall** metrics, was used to find the best tradeoff between these two quantities:

$$F1‐\text{score}=2\;\frac{\mathrm{precision}\cdot \mathrm{recall}}{\mathrm{precision}+\mathrm{recall}}.$$

Indeed, it is a complex matter to simultaneously increase **precision** and **recall** once they have reached a certain value. Thus, **F1‐score** rewards models that have similar **precision** and **recall** values rather than models that decrease one of them to reach a value near 1.0 with the other [12].

Finally, the last metric was the **AUC**, representing the area under the ROC curve defined by the plot of the **recall** (true positive rate) versus the false positive rate ($\mathrm{FPR}=\frac{\mathrm{FP}}{\mathrm{TN}+\mathrm{FP}}$). This area represents the inherent ability of the model to discriminate between healthy and diseased population [13].

### Push‐off classification versus athlete classification

The algorithms were trained to recognize knee injury sequelae from push‐offs. Nevertheless, to get the model prediction with such sequelae for each athlete, all push‐off predictions belonging to one athlete have been gathered. Then, the performance metric scores for athlete classification were the average of the metric scores of all athletes' push‐offs. The prediction score for athlete classification was the number of push‐offs classified as ‘presence of knee injury sequelae’ divided by the number of push‐offs.

### SHAP method

Despite their success, ML algorithms such as neural networks and gradient boosting methods are often compared to ‘black boxes’ because even the users of these algorithms can find it difficult to explain why the algorithm made a particular decision [30]. To address this issue, explainable artificial intelligence has been developed to make the decision‐making of ML algorithms more understandable to humans. The chosen technique for the present study was SHAP which stands for the SHAP and originated from the game theory [30]. The SHAP were computed by carefully perturbing input features and seeing how these changes could impact the model prediction. The Shapley value of a given feature was then calculated as its average marginal contribution to the overall model score. SHAP were computed with respect to a comparison or background group which served as a ‘baseline’ for the explanation. Thus, ML algorithms based on SHAP can be used to explain both a particular prediction and general outcomes of a model [30].

### Statistical analysis

For each train‐test split (*n* = 1000), each metric (accuracy, precision, recall, F1‐score, AUC) was computed for each model and data set. Two‐way analysis of variance was conducted to determine the effect of model selection (DT, XGB and MLP) and used data set (with or without psychological variables) on classification performances, that is, AUC values. When significant differences were identified, Bonferroni post hoc tests were used to identify pairwise differences. Such analysis was done for push‐off classification and for athlete classification. The threshold of significance was set at *p* < 0.05.

## RESULTS

### Results on push‐offs

Performances related to the push‐off classifications are depicted in Table 3. On average (first part of Table 3), over the thousand cross‐validations, higher AUC values were obtained when accounted for the psychological attributes (F_1,5994_ = 71.7, *p* < 0.001). Also, a main effect of model selection has been found (F_2,5994 _= 91.4, *p *< 0.001) with higher AUC values from MLP compared to XGB or DT (*p *< 0.001). XGB exhibited higher AUC values than DT (*p *< 0.001). The second part of Table 3 presents the best performance of each model for a specific train‐test split (i.e., the number indicated in the last column of the table). In such specific conditions, it appeared that DT showed the best performances compared to XGB and MLP when accounting for the psychological attributes in almost all the metrics.

**Table 3:** Performance metrics from push‐off classifications.

Attributes selected for training	Model	Accuracy	Precision	Recall	F1‐score	AUC	Test sample
Without psychological attributes	DT	0.56 (0.09)	0.46 (0.14)	0.41 (0.17)	0.42 (0.14)	0.54 (0.10)	Overall mean
	XGB	0.56 (0.10)	0.47 (0.14)	0.45 (0.17)	0.45 (0.14)	0.55 (0.10)	Overall mean
	MLP	0.63 (0.07)	0.46 (0.35)	0.24 (0.24)	0.29 (0.26)	0.57 (0.09)	Overall mean
With psychological attributes	DT	0.58 (0.11)	0.48 (0.17)	0.41 (0.18)	0.43 (0.16)	0.55 (0.11)	Overall mean
	XGB	0.59 (0.10)	**0.51** (0.16)	**0.46** (0.18)	**0.47** (0.15)	0.57 (0.11)	Overall mean
	MLP	**0.65** (0.08)	**0.51** (0.33)	0.31 (0.26)	0.36 (0.27)	**0.60** (0.10)	Overall mean
Without psychological attributes	DT	0.84	0.81	0.77	0.79	0.83	845
	XGB	0.82	0.75	0.87	0.80	0.83	731
	MLP	0.85	0.84	0.77	0.80	0.83	883
With psychological attributes	DT	**0.95**	0.99	**0.88**	**0.93**	**0.94**	461
	XGB	0.90	0.86	0.87	0.87	0.89	643
	MLP	0.93	**1.0**	0.82	0.90	0.91	356

*Note*: First part of the table: Averages (and standard deviations) of each metric for each algorithm over the thousand cross‐validations made on each data set. The second part of the table: Best metrics achieved for one specific model training. Bold values represent the best metric score obtained.

Abbreviations: AUC, area under the curve; DT, decision tree; MLP, multilayer perceptron; XGB, extreme gradient boosting.

### Results on athletes

Performances related to the athlete classifications are depicted in Table 4 and were quite similar to those related to push‐off classifications. On average (first part of Table 4), higher AUC values were obtained when accounted for the psychological attributes (F_1,5994_ = 54.0, *p* < 0.001). Also, a main effect of model selection has been found (F_2,5994 _= 96.4, *p* < 0.001) with higher AUC values from MLP compared to XGB or DT (*p* < 0.001). XGB exhibited higher AUC values than DT (*p* < 0.001). The second part of the Table 4 presents the best performance of each model for a specific train‐test split. In such specific conditions, it appeared that DT and MLP showed the best performances in comparison with XGB and when accounted for the psychological attributes in all the metrics.

**Table 4:** Performance metrics from athlete classifications.

Attributes selected for training	Model	Accuracy	Precision	Recall	F1‐score	AUC	Test sample
Without psychological attributes	DT	0.56 (0.10)	0.45 (0.16)	0.42 (0.18)	0.42 (0.15)	0.54 (0.11)	Overall mean
	XGB	0.56 (0.10)	0.45 (0.15)	0.45 (0.18)	0.44 (0.14)	0.54 (0.11)	Overall mean
	MLP	0.64 (0.07)	0.43 (0.38)	0.25 (0.25)	0.29 (0.26)	0.58 (0.09)	Overall mean
With psychological attributes	DT	0.57 (0.11)	0.47 (0.18)	0.41 (0.19)	0.42 (0.16)	0.55 (0.11)	Overall mean
	XGB	0.59 (0.11)	**0.49** (0.16)	**0.46** (0.18)	**0.46** (0.15)	0.57 (0.11)	Overall mean
	MLP	**0.66** (0.08)	0.48 (0.34)	0.32 (0.26)	0.36 (0.27)	**0.60** (0.11)	Overall mean
Without psychological attributes	DT	0.90	0.88	0.88	0.88	0.90	845
	XGB	0.75	0.67	0.75	0.71	0.75	731
	MLP	0.85	0.86	0.75	0.80	0.83	883
With psychological attributes	DT	**0.95**	**1.0**	**0.88**	**0.93**	**0.94**	461
	XGB	0.90	0.88	0.88	0.88	0.90	643
	MLP	**0.95**	**1.0**	**0.88**	**0.93**	**0.94**	356

*Note*: First part of the table: Averages (and standard deviations) of each metric for each algorithm over the thousand cross‐validations made on each data set. The second part of the table: Best trained models for each data set for athlete classification. Bold values represent the best metric score obtained.

Abbreviations: AUC, area under the curve; DT, decision tree; MLP, multilayer perceptron; XGB, extreme gradient boosting.

**Figure 2:**
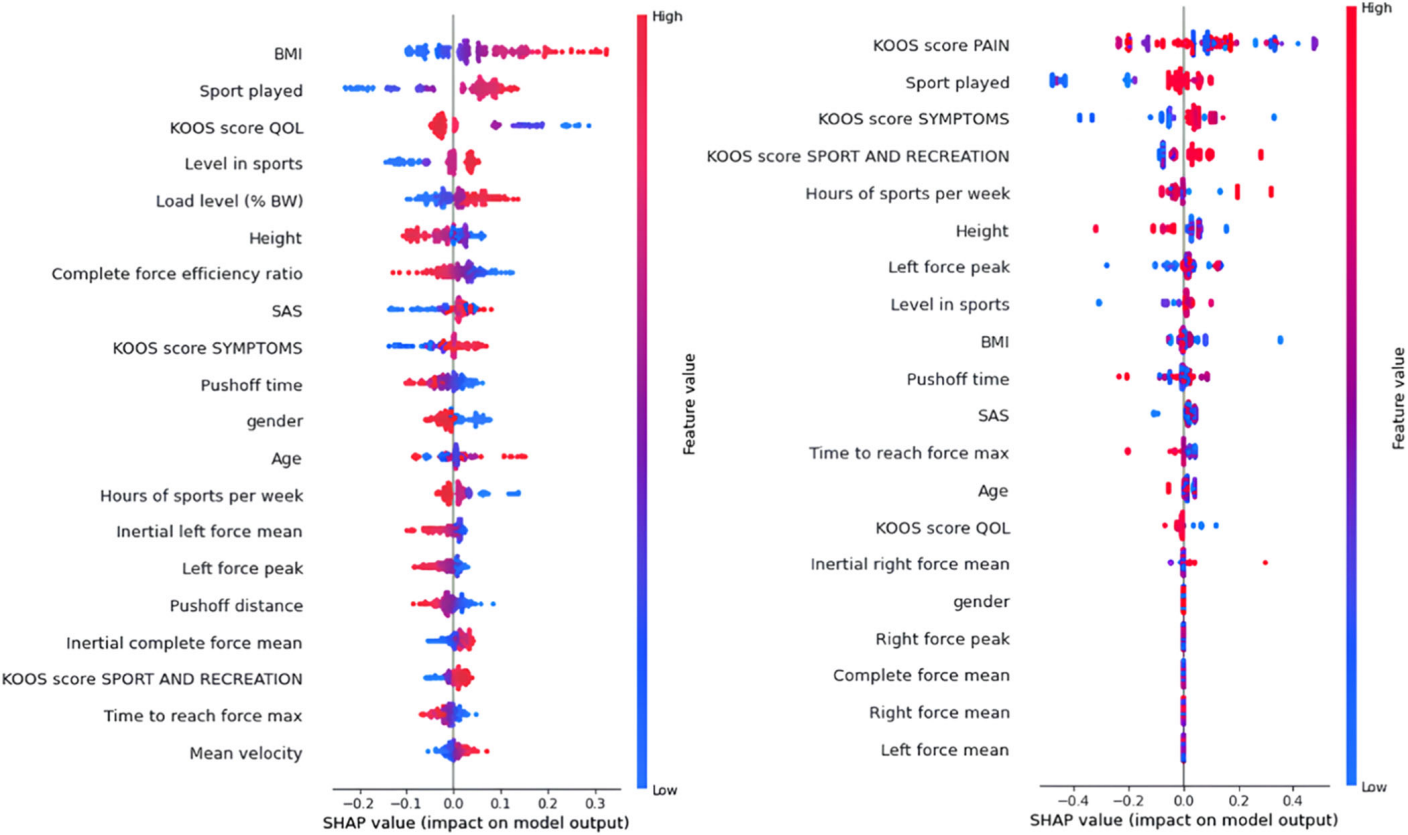
SHAP graph of the most influential variables for MLP (left panel) and DT (right panel) models with psychological attributes. From top to bottom are the attributes ranking from the most to the less influential. Points on the right increased the prediction score of knee injury sequelae whereas points on the left decreased this prediction score. Red values are for high variable values and blue values are for low variable values. BMI, body mass index; DT, decision tree; KOOS, Knee Injury and Osteoarthritis Outcome Score; MLP, multilayer perceptron; QOL, quality of life; SAS, Sport Anxiety Scale; SHAP, shapley value method.

Figure 2 presents the variables among those summarized in Table 1 that influenced the most models' predictions for the two best athlete classification models: DT and MLP with psychological attributes (second part of Table 4). These features are general risk factors for the presence of knee injury sequelae based on the athletes in our data set.

According to the best DT model (on the right of Figure 2), an athlete practicing more than 8 h a week with high scores in sport and recreation, and symptoms subsections of the KOOS (meaning good values) is more likely to have knee injury sequelae. For the best MLP model (on the left of Figure 2), male athletes with high body mass index (BMI), practicing ski, volleyball, basketball or gymnastics at a high level and with low scores in the ‘quality‐of‐life’ subsection of the KOOS are more likely to present knee injury sequelae. In addition to general risk factors identified in our data set, the SHAP technique allowed us to explain individual predictions. For instance, we used this method to elucidate the most influential factors for an athlete who had experienced a partial ACL tear exactly 2 years prior to the test and who was correctly classified by both models as having knee injury sequelae. For the DT model, the key factors included low scores in every subsection of KOOS, the sport played (football) and the athlete's long push‐off time. For the MLP model, the major influencing factors were low KOOS scores in the quality of life (QOL) and symptoms sections, the type of sport played (football) and the athlete's BMI.

## DISCUSSION

Over a thousand train‐test splits, MLP and XGB models were on average better than DT. Moreover, regardless of the ML model and the type of classification, adding psychological variables (KOOS, SAS) to informative physical and biomechanical attributes increased prediction performances. This conclusion was strongly supported by the results from the SHAP analysis (Figure 2) with psychological variables, particularly the KOOS subsections scores (Pain, QOL, Symptoms, Sport and Recreation), being predominantly at the top of the list, ahead of biomechanical, physical and informational variables. Differences in feature hierarchy between models are a common issue as noted by Saarela et al. [28] who found that different features of importance were detected by different methods for injury prediction in running. The present results were in agreement with the conclusions of Andrade et al. [2] who found in their meta‐analysis that KOOS subsections scores were discriminant factors in recognizing return‐to‐play and nonreturn‐to‐play athletes after ACL reconstruction. In their meta‐analysis, they observed that the return‐to‐play criteria were study‐specific: some studies considered that patients were cleared for RTP after just a range of motion tests, others after a specific rehabilitation programme and finally other studies waited for patients to play at the same level than before. However, regardless of the return‐to‐play criteria, the conclusion remained consistent with psychological attributes being more influential than biomechanical ones.

Focusing on models trained with psychological attributes, both MLP and DT achieved the same single best performances but on two different specific train‐test splits. Finally, no matter the train‐test split, the chance to obtain better results was higher with MLP than with DT as hypothesized because of the better computational power of MLP models [16]. As a consequence, MLP seemed to be the most suitable ML algorithm for classifying push‐offs and subsequently, for classifying athletes in relation to the presence of knee injury sequelae. The average AUC presented in this study for MLP models (0.60 ± 0.10) was slightly lower than the best AUC presented by Jauhiainen et al. [17] in a similar study (0.63 ± 0.02). However, we performed one thousand train‐test splits to limit the selection bias contrary to the hundred made by these authors. One can suggest that the differences in mean and standard deviation were due to the larger number of train‐test splits performed in the current study. Indeed, MLP models sometimes had very fluctuating performance metrics (see standard deviations of **precision**, **recall** and **F1‐scores**). As a result, the more training and testing we did, the greater the possibility of obtaining either very poor or very strong results, leading to higher standard deviation and differences in mean. As a consequence, it is not possible to draw strong conclusions from the results obtained on one random split, especially with such a small data set. This variability was also highlighted by Ruddy et al. [27] who found AUC ranging from 0.24 to 0.92 on 10 cross‐validations for injury prediction over 186 Australian football players. This bias can be explained by the highly multifactorial and athlete‐dependent aspects of knee injuries, but also by the horizontal squat jump test we chose to perform. Indeed, the supine position to develop a maximum power on horizontal push‐offs could be disturbing for some athletes no matter their injury background. To reduce this bias, increasing the cohort size up to 500 athletes would provide more training examples for the ML models. However, as this is a challenging task, finding a balance between cohort size and the number of train‐test splits performed would be an alternative approach. As far as we are concerned, we chose to do a thousand of them as a tradeoff between running time (10 h on our computer) and all train‐test split possibilities (more than a billion).

The present study focused on identifying sequelae of knee injury instead of following up with athletes and monitoring injury occurrence as previously done. For instance, Jauihainen et al. [17] tested a large population during the preseason and then monitored the occurrence of knee injuries during the season to build their database. Despite this difference in methodological approach, the performances of the present algorithms were quite similar to those of Jauihainen et al. [17]. This suggested that our models worked well in predicting knee injury while all of our athletes were clinically allowed to practice sports and thus made predictions based on subtle differences in physical performance. Overall, focusing on knee injury sequelae allowed us to emphasize preventing the recurrence of knee injuries by optimizing the return‐to‐play process. Specifically, the SHAP technique could provide the variables that most influenced the model's prediction for a particular athlete. For instance, it was applied to an individual who suffered a partial ACL tear 2 years before the test and was correctly classified by both MLP and DT. Both models exhibited several similar risk factors for this participant though ranked differently: KOOS QOL score (1st for MLP, 4th for DT) and generally all subsection scores of the KOOS were among the twenty most important features alongside the push‐off time and the time to reach peak propulsive force. Nonetheless, for the MLP method, there were more biomechanical attributes on which this athlete could work than for DT (mean velocity, left mean inertial force, right and left peak force). As a consequence, the ranking of influential variables obtained with SHAP for individuals with a history of knee injury who were correctly classified could help athletes, trainers and physiotherapists identify key variables to focus on when building a training or rehabilitation programme. It could also serve as another indicator to help determine the appropriate time for the athlete's RTP, thereby reducing the risk of knee injury relapse on the same or contralateral leg.

While the present study achieved varying AUC values through a thousand train‐test splits, highlighting the challenge of generalizing ML models in rehabilitation [27], it emphasized the importance of individualization in injury prediction, aligning with the need expressed by previous authors [34]. Practical applications based on the present findings include the potential to tailor training programmes according to a multitude of psychological (e.g., mental health questionnaire scores) and biomechanical factors (e.g., force, velocity, power…). Clinically, it could help to individually optimize the time of RTP by providing new information to professionals responsible for making this decision.

Despite the valuable insights gained from this study, several limitations must be acknowledged. One limitation was the small number of subjects included in this study. This may have influenced the performance of the ML models and their generalization. This population was also very heterogeneous with individuals practicing different sports, playing at different levels and with different knee injuries. Expanding the data set considerably would allow for a more precise investigation of specific injuries and sports without compromising performance. Another limitation could be the use of the horizontal force–velocity test which was not a dedicated test used in clinical routine to assess knee injury or recovery. Addressing these limitations and adding data obtained from other physical tests (e.g., triple hop tests, agility tests, …) would enhance performance and the impact of this research.

## CONCLUSIONS

The present study suggested that psychological factors play a more important role than biomechanical factors in ML model recognition of knee injury in athletes. Additionally, the computing stability of MLP makes it the reference for classification tasks as it is now possible to interpret results from these neural networks. Nevertheless, future studies should aim to expand the sample size and integrate psychological data alongside results of other physical evaluations to further validate this approach in clinical routine.

## AUTHOR CONTRIBUTIONS

*Study conception and design*: Clément LIPPS LENE, Julien Frere and Thierry Weissland. *Analysis and interpretation of results*: Clément LIPPS LENE. *Draft manuscript preparation*: Clément LIPPS LENE. All authors reviewed the results and approved the final version of the manuscript.

## CONFLICT OF INTEREST STATEMENT

The authors declare no conflict of interest.

## ETHICS STATEMENT

An Ethics Review Committee (CERSTAPS: IRB00012476‐2022‐21‐01‐149) approved the study. All individuals gave written consent before participating in the study. Written informed consent was obtained from a legally authorized representative for anonymized subject information to be published in this article.

## Data Availability

The data sets generated and/or analyzed during the current study are not publicly available because no political agreement was found for the public sharing of data between universities where data were acquired. Nonetheless, data are available from the corresponding author upon reasonable request.
